# The limit of resistance to salinity in the freshwater cyanobacterium *Microcystis aeruginosa* is modulated by the rate of salinity increase

**DOI:** 10.1002/ece3.6257

**Published:** 2020-04-14

**Authors:** Ignacio José Melero‐Jiménez, Elena Martín‐Clemente, María Jesús García‐Sánchez, Elena Bañares‐España, Antonio Flores‐Moya

**Affiliations:** ^1^ Departamento de Botánica y Fisiología Vegetal Facultad de Ciencias Universidad de Málaga Málaga Spain

**Keywords:** adaptation, Cyanobacteria, environmental deterioration rate, ratchet protocol, salinity

## Abstract

The overall mean levels of different environmental variables are changing rapidly in the present Anthropocene, in some cases creating lethal conditions for organisms. Under this new scenario, it is crucial to know whether the adaptive potential of organisms allows their survival under different rates of environmental change. Here, we used an eco‐evolutionary approach, based on a ratchet protocol, to investigate the effect of environmental change rate on the limit of resistance to salinity of three strains of the toxic cyanobacterium *Microcystis aeruginosa.* Specifically, we performed two ratchet experiments in order to simulate two scenarios of environmental change. In the first scenario, the salinity increase rate was slow (1.5‐fold increase), while in the second scenario, the rate was faster (threefold increase). Salinity concentrations ranging 7–10 gL^‐1^ NaCl (depending on the strain) inhibited growth completely. However, when performing the ratchet experiment, an increase in salinity resistance (9.1–13.6 gL^‐1^ NaCl) was observed in certain populations. The results showed that the limit of resistance to salinity that *M. aeruginosa* strains were able to reach depended on the strain and on the rate of environmental change. In particular, a higher number of populations were able to grow under their initial lethal salinity levels when the rate of salinity increment was slow. In future scenarios of increased salinity in natural freshwater bodies, this could have toxicological implications due to the production of microcystin by this species.

## Graphical abstract

We used an eco‐evolutionary approach based on a ratchet protocol to investigate the effect of environmental change rate on the limit of resistance to salinity of three strains of the toxic cyanobacterium *Microcystis aeruginosa*. The limit of resistance to salinity that *M. aeruginosa* strains were able to reach depended on the strain and on the rate of environmental change.

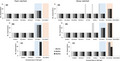

## 1 INTRODUCTION

The response of organisms to increasing selection pressure initially occurs through the modification of gene expression during a period extending from hours to few days (i.e., acclimation; Bradshaw & Hardwick, 1989; Fogg, 2001). However, the selective pressure could keep increasing exceeding the physiological limits of the organism. As increasing selection pressure threatens population extinction, recombination between and *de novo* mutation within individually selected genotypes becomes necessary for population survival (i.e., adaptation, Belfiore & Anderson, 2001; Borowitzka, 2018). Apart from the species’ existing genetic pool, it has been suggested that adaptation could also depend on the rate of environmental change (Ashander, Chevin, & Baskett, 2016; Collins & de Meaux, 2009; Collins, Meaux, & Acquisti, 2007). Thus, a gradual increment of stress favors population persistence because the allele or alleles responsible for survival can be selected under sublethal stress conditions, allowing a gradual adaptation (Bell, 2013; Bell & Collins, 2008; Bell & Gonzalez, 2011; Carlson, Cunningham, & Westley, 2014; Collins & de Meaux, 2009; Gonzalez & Bell, 2013; Kirkpatrick & Peischl, 2012; Lindsey, Gallie, Taylor, & Kerr, 2013; Perron, Gonzalez, & Buckling, 2008; Samani & Bell, 2010). Besides, under gradual stress, population survival will maintain a high population size and, as a consequence, there would be with lower chance of beneficial alleles being lost to drift and a higher probability for beneficial alleles to recombine together into well‐adapted genotypes (Lande, Engen, & Saether, 2003). In contrast, a sudden environmental change would cause a strong reduction of population fitness, leading to a sudden decrease in the number of individuals (lower genetic diversity) and thereby reduce the chance for resistant mutants to appear in the population (Bell & Collins, 2008; Collins et al., 2007). Consequently, the limit of resistance of a population would be determined by the rate of environmental change, as it has been proved, for example, for antibiotic resistance in *E. coli* (Perron et al., 2008,2006). In addition, several studies have shown that the probability of evolutionary rescue (i.e., the possibility that natural populations exposed to lethal conditions may adapt through natural selection) is greater when the rate of environmental change is slow rather than fast (Bell & Gonzalez, 2011; Gonzalez & Bell, 2013; Killeen, Gougat‐barbera, Krenek, & Kaltz, 2017; Lindsey et al., 2013; Perron et al., 2008; Samani & Bell, 2010). For example, Lindsey et al. (2013) observed that the resistance of an *E. coli* population is determined by its historical sequence of environmental conditions.

Salinity values are rising in many freshwater bodies worldwide due to climate change and human activities, such as agriculture or water extraction, decreasing the phreatic level in coastal areas, leading to seawater intrusion (Cañedo‐Argüelles et al., 2013, 2016; Kaushal et al., 2018; Kundzewicz, 2008; Nielsen, Brock, Rees, & Baldwin, 2003; Olson, 2018; Werner & Gallagher, 2006; Williams, 2001). For this reason, it is interesting to perform long‐term experiments in order to know the limit of resistance of freshwater organisms to salinity increase. Moreover, to make future predictions under this new scenario of increased salinity in freshwater ecosystems, the knowledge of how different rates of salinity increase modulate the limit of resistance is crucial.

In a previous study, using an eco‐evolutionary approach, we demonstrated that the selection of spontaneous mutants allowed the survival of the freshwater cyanobacterium *Microcystis aeruginosa* (Kützing) Kützing when exposed to lethal salinity concentrations (Melero‐Jiménez, Martín‐Clemente, García‐Sánchez, Flores‐Moya, & Bañares‐España, 2019). Moreover, we determined the limit of resistance to this selective agent at a single rate of salinity increase (Melero‐Jiménez et al., 2019). However, the rate of environmental change (salinity increment) could determine the limit of resistance to salinity in this species. It must be highlighted that most of the studies focused on the effect of increased salinity on *M. aeruginosa* assessed the performance of cells after only a few generations (less than ten) submitted to high salinity (Martínez de la Escalera et al., 2017; Miller et al., 2010; Rosen et al., 2018; Orr, Jones, & Douglas, 2004; Otsuka et al., 1999; Prinsloo & Pieterse, 1994; Tonk, Bosch, Visser, & Huisman, 2007; Verspagen et al., 2006; Zhang, Xu, & Xi, 2013). Consequently, these results could be interpreted as evidence of cell regulation and acclimation (Borowitzka, 2018). On the contrary, only a few studies have been performed submitting cell populations to a selective agent for a high number of generations. In these studies, a fluctuation analysis design based on Luria and Delbrük (1943) was applied indicating that selection of new genotypes of *M. aeruginosa* (i.e., adaptation) occurred under exposition to different selective agents (Fernández‐Arjona et al., 2013; García‐Villada et al., 2004; Huertas, Rouco, López‐Rodas, & Costas, 2011; López‐Rodas et al., 2007; Rouco et al., 2014), including high salinity (Melero‐Jiménez et al., 2019). However, we want to explore two more questions; could the limit of resistance to a selective agent be different depending on the rate of environmental deterioration? and would it affect different strains the same?

The aim of the present work was to analyse the differential adaptive potentials of three strains of the cyanobacterium *M. aeruginosa* subjected to either a slow or a rapid increment in salinity. First, we performed a ratchet experiment to analyse the effect of two different environmental deterioration rates on the limit of resistance of *M. aeruginosa* to salinity. Secondly, a complementary experiment was performed to disentangle whether the observed limit of resistance to salinity was due to acclimation or to the selection of new genetic variants.

## 2 METHODS

### 2.1 Experimental organisms and culture conditions

Three strains of the cyanobacterium *M. aeruginosa* (Ma1Vc, Ma5Vc, and MaAVc) were provided by the Algal Culture Collection of the Genetics Laboratory, Veterinary School, Complutense University (Madrid, Spain). All the strains were isolated in April 2011 from the Valmayor reservoir (Madrid, Spain), a freshwater reservoir where conductivity is less than 200 μScm^‐1^ (Carrasco et al., 2006). Therefore, it was assumed that the strains had no previous evolutionary history of salinity exposure. Culture conditions were similar to those described in our previous studies (Bañares‐España et al., 2016, Carrillo et al., 2003; Costas et al., 2007; Fernández‐Arjona et al., 2013; López‐Rodas et al., 2007, 2009; Huertas, Rouco, López‐Rodas, & Costas, 2010; Huertas et al., 2011; Rouco et al., 2014; García‐Villada et al., 2004; Martín‐Clemente, Melero‐Jiménez, Bañares‐España, Flores‐Moya, & García‐Sánchez, 2019; Melero‐Jiménez, et al., 2019). Cultures were grown axenically in 250 mL ventilated cell culture flasks sealed with filter screw‐caps (Greiner; Bio‐One), containing 60 mL of half‐diluted BG11 medium (Sigma‐Aldrich Chemie). Previously, it was confirmed that the strains’ growth rates were similar to those in full strength BG11 (data not shown). Cultures were incubated at 20°C under a continuous photon flux density of 60 μmolm^‐^^2^ s^−1^ over the waveband 400–700 nm provided by cool‐white fluorescent lamps. They were maintained in mid‐log exponential growth by weekly serial transfers of inocula to fresh medium.

### 2.2 Toxicity test: effect of NaCl on growth rate

In order to determine the lethal NaCl dose for each strain, a toxicity test was performed. BG11‐50% culture media containing 0, 2, 4, 8, 16, and 24 gL^‐1^ NaCl were prepared. Each experimental culture was inoculated with 6 × 10^5^ wild‐type strain cells from mid‐log exponentially growing cultures. Three replicates of each NaCl concentration were prepared. The effect of NaCl was estimated by calculating the growth rate (*m*) in mid‐log exponentially growing cells, using the equation (Crow & Kimura, 1970):

(1) $$m\;=\;\log_{e}\left(N_{t}/N_{0}\right)/t,$$

where *t* = 7 days, and *N*_t_ and *N*_0_ are the cell number at the end and at the start of the experiment, respectively. Experimental and control cell numbers (CN, units in cells mL^‐1^) were determined by the linear fit CN = A_750_ × 1.11 × 10^7^ (*r* ^2^ = 0.984, *n* = 18), where A_750_ is the absorbance of the cell culture at λ = 750 nm measured in a spectrophotometer (Selecta UV‐2005, Spain). The number of cells was counted blind (i.e., the person counting did not know the identity of the tested sample) using a hemocytometer (BLAUBRAND^®^ Neubauer improved, Germany). The number of samples in each case was determined using the progressive mean procedure (Williams, 1977), which assured a counting error less than 5%. Lethal doses were estimated by linear fit of *m* as a function of NaCl concentration.

### 2.3 Experimental design: ratchet protocol

**FIGURE 1:**
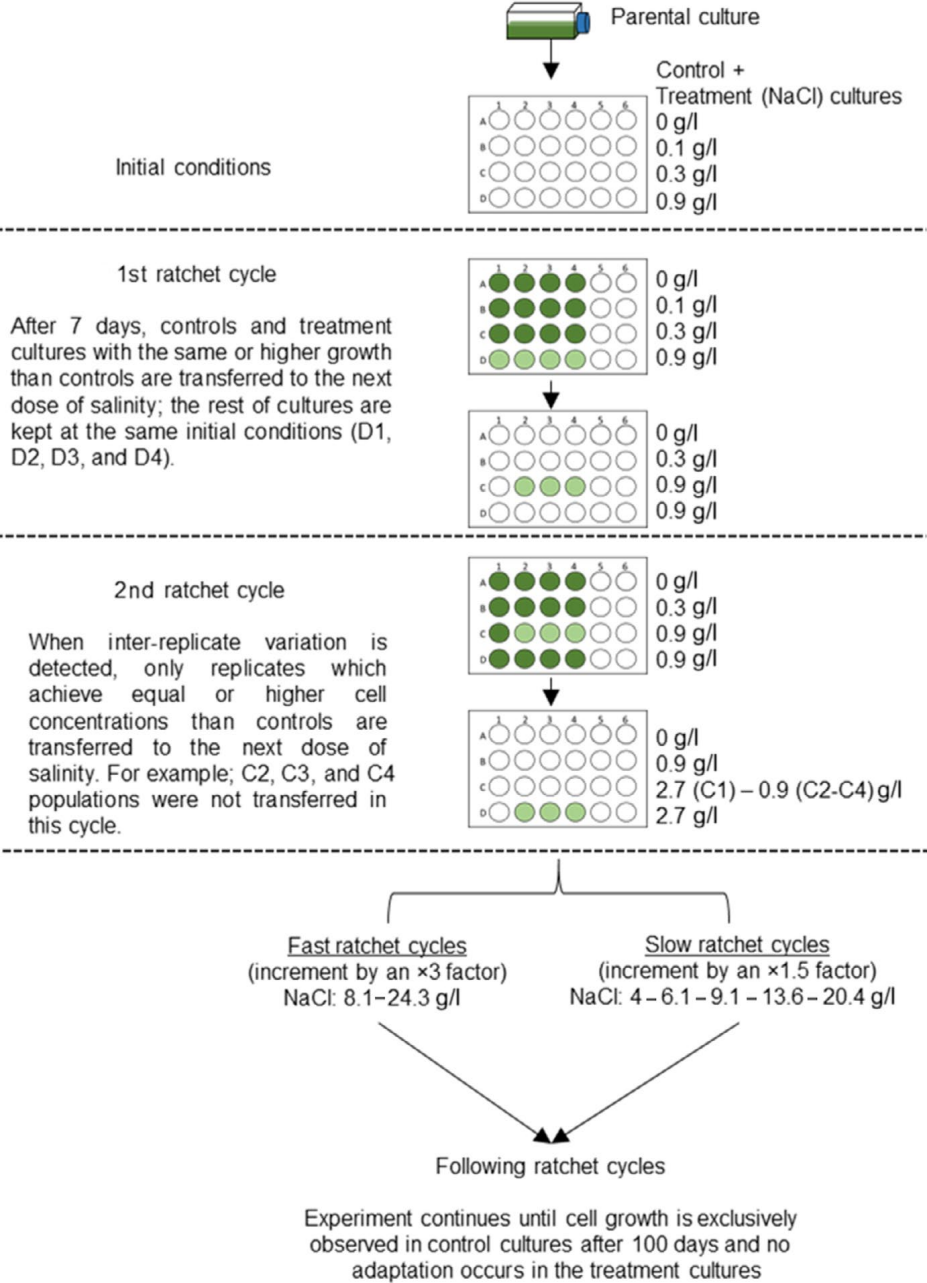
Schematic representation of the ratchet experimental design. Four replicates (1–4 wells) of the control cultures (A wells) and four replicates of cultures at each of the three initial NaCl doses (B‐D wells, respectively) were incubated at each ratchet cycle. Each culture was transferred to the next, threefold higher NaCl concentration, when the same net growth as the control cultures was observed. Cultures that did not grow as controls were maintained at the same salinity. A new ratchet cycle began when the treatment cultures were transferred to the next concentration. When the cultures reached the concentration of 2.7 gL^‐1^ NaCl, a different rate of environmental deterioration was applied. From this point, NaCl concentrations increased 1.5‐fold or threefold in the slow and fast ratchet, respectively. The limit of resistance corresponds to the maximum salinity at which net growth was detected

In order to analyze the effects of environmental deterioration rate on the limit of resistance to salinity of each of the three strains of *M. aeruginosa*, a ratchet experiment was performed as described by Huertas et al. (2010, 2011), Rouco et al. (2014), Melero‐Jiménez et al. (2019), and Martín‐Clemente et al. (2019). Two ratchet experiments were carried out to simulate fast and slow environmental deterioration rates (Figure 1). It must be highlighted that the ratchet protocol is designed to balance a strong selection pressure and a population size large enough to assure the occurrence of new mutations that confer resistance. In short, experimental cultures were inoculated with an elevated cell density and exposed to increasing NaCl levels. In parallel, four control cultures (without NaCl) were started at time zero, and all experimental cultures were quadruplicated at each of the three initial NaCl concentrations (described below). Cultures were grown separately in wells containing 2 mL of culture medium inoculated with 3 × 10^5^ cells in a 24 well‐plate. This number of cells was considered large enough to ensure the occurrence of a large final population after applying a salinity increase. Three initial NaCl doses were used (0.1, 0.3, and 0.9 gL^‐1^), and the NaCl concentration was increased threefold at each ratchet cycle and at each initial dose. Cultures were kept under the experimental salinity levels for 7 days prior to observation, allowing the development of a large final population with enough cell density to increase the chances for the appearance of resistant cells. At this time, cell concentrations in controls were compared with those of the experimental cultures containing NaCl. The experimental cultures were transferred to a higher salinity when their cell concentrations were similar to, or higher than that in control cultures. The experimental cultures showing a cell concentration lower than the control cultures were not transferred. After comparing treatments with controls, a new ratchet cycle began; control and experimental cultures were again inoculated with cell concentrations identical to those used during the first ratchet cycle. When the cultures of the three ratchet experiments reached a concentration of 2.7 gL^‐1^ NaCl, that is, before reaching the lethal dose (previously determined, see *2.2. Toxicity test: effect of NaCl on growth rate)*, an additional NaCl concentration increase was applied (×1.5 factor) to establish a slower deterioration rate (Figure 1), maintaining the initial × 3 factor for the fast environmental deterioration condition. Thus, succeeding ratchet cycles were 8.1 and 24.3 gL^‐1^ NaCl on fast ratchet, and 4, 6.1, 9.1, 13.6, and 20.5 gL^‐1^ NaCl on slow ratchet. Finally, we established the limit of resistance as the highest salinity that allowed cell growth. The number of generations (*g*) during the ratchet cycles was computed in accordance with Novick and Szilard (1950):

(2) $$g\;=\;\log_{e}\left(N_{t}/N_{0}\right)/\log_{e}2$$

where *N*_t_ and *N*_0_ were the number of cells at time *t* (when the cell density in the culture was similar to that the control) and time 0, respectively.

### 2.4 Acclimation versus adaptation

**FIGURE 2:**
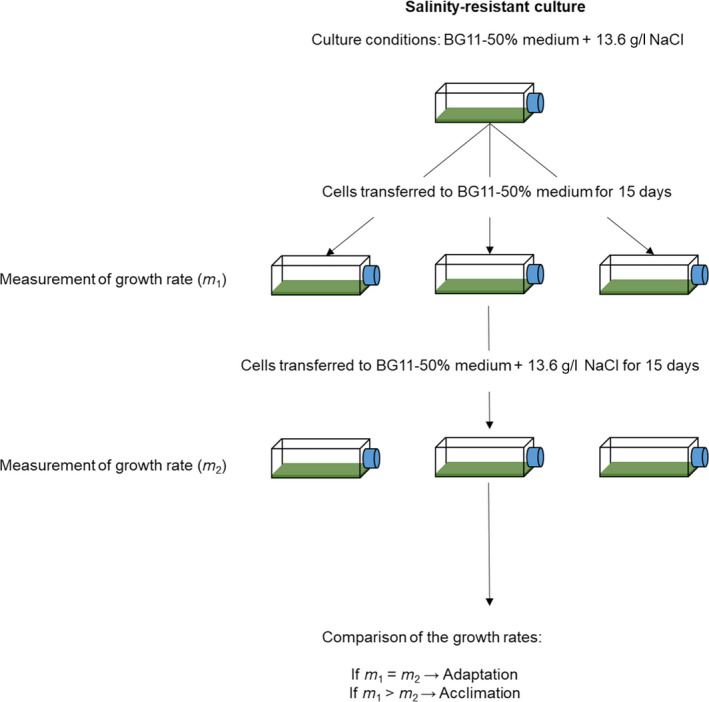
Experimental design to disentangle acclimation versus adaptation of the resistant cells derived from the ratchet experiment. In the first step, the cells adapted to the highest salinity level after running the ratchet experiment (9.1 gL^‐1^ NaCl for strain Ma1Vc, and 13.6 gL^‐1^ NaCl for strains Ma5Vc and MaAVc, as shown in the figure) were transferred for 15 days to BG11‐50% medium (without NaCl additions) and the growth rate (*m*_1_ *)* was determined. Then, the cells were transferred for another 15 days to BG11‐50% supplemented with the corresponding NaCl concentration, and the growth rates (*m*_2_) were computed again. Adaptation can be recognized by a similar *m*_1_ and *m_2_* values whereas if *m*_2_ is significantly lower than *m*_1_, it can be inferred that resistance was mainly due to acclimation

The ratchet protocol is not designed to disentangle whether the observed limit of resistance of a microorganism to a selective agent results from a process of acclimation or adaptation. For this reason, in order to disentangle between both mechanisms, the approach followed by Rouco et al. (2014) was applied (Figure 2). This complementary experiment is based on that, at least for bacteria, acclimation effects processes, including epigenetic events, can span two‐three generations (Bennet & Lenski, 1997).

Four independent cultures of the salinity‐resistant cells of each strain derived from the ratchet experiment were founded with initial inocula of 6 × 10^5^ cells in 20 mL BG11‐50% medium, without any NaCl addition. Cultures were incubated for 15 days (~4 generations) in the same growth conditions as described before. Then, the *m* values were determined as explained above (see the section 2.2 *Toxicity test: effect of NaCl on growth rate).* After that, the cells were transferred for another 15 days to BG11‐50% medium supplemented with 9.1 gL^‐1^ NaCl for strain Ma1Vc, and 13.6 gL^‐1^ NaCl for strains Ma5Vc and MaAVc (these values were the highest at which the cells showed growth in the previous ratchet experiment), and the *m* value was measured again (Figure 2). The *m* values of wild‐type cells, and the *m* values of the salinity‐resistant cells grown in BG11‐50% medium (*m*_1_) and in BG11‐50% supplemented with the highest salinity level (*m*_2_), were compared using the nonparametric Kruskal–Wallis *H* test. When significant differences were found, the Mann–Whitney test, with the Bonferroni correction, was applied. Data analysis was performed in R statistical environment (R Core Team, 2016). We consider that adaptation, although other processes cannot be excluded, occurs when *m_1_* and *m_2_* values are similar, that is the growth rate of the salinity‐resistant cells do not show significant differences when grown at their ancestral conditions (without NaCl additions) and in the presence of high NaCl concentrations (at their limit of resistance). In contrast, we consider that acclimation occurs when the *m* value measured after salinity exposure is lower than that measured in the absence of NaCl.

## 3 RESULTS

### 3.1 Toxicity test: effect of NaCl growth rate

**FIGURE 3:**
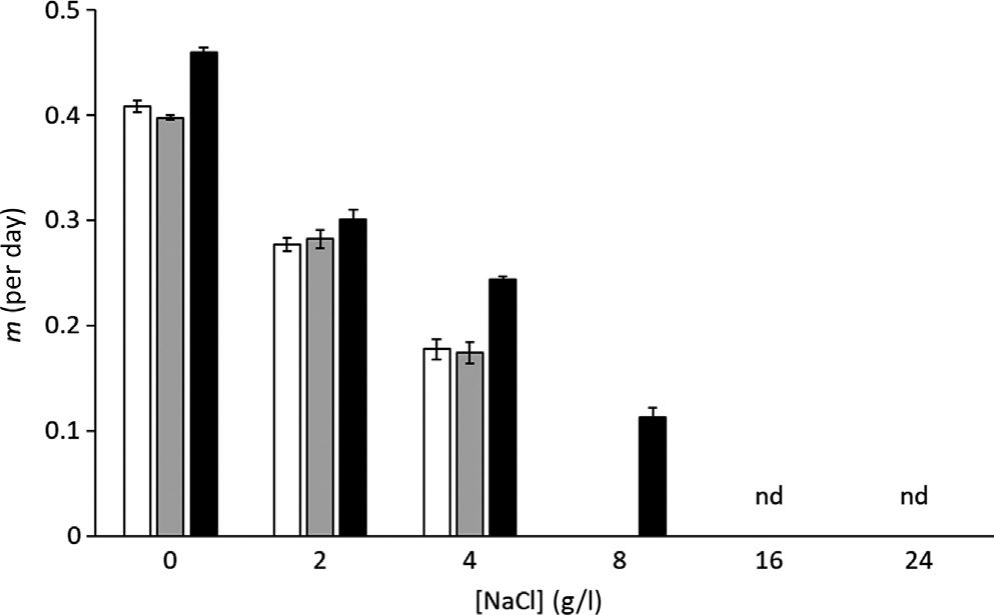
Effect of NaCl on growth rate (*m*) of Ma1Vc (white bars), Ma5Vc (gray bars) and MaAVc (black bars) strains of *Microcystis aeruginosa*. The *m* values were computed after 7 days of exposure to NaCl in mid‐log exponentially growing culture cells. Data are mean ± *SD* (*n* = 3); nd, nondetectable growth for all three strains

The exposure to increasing salinities (from 0 to 24 gL^‐1^ NaCl) had a toxic effect on wild‐type cells of the three strains of *M. aeruginosa*, *m* being undetectable at concentrations ≥ 8 gL^‐1^ NaCl depending on the strains (Figure 3). Lethal doses were estimated by linear fit of *m* as a function of NaCl concentration:

$$m_{\text{Ma1Vc}}=\;-0.057\;\times \;\left[{\text{g L}}^{-1}\text{NaCl}\right]\;+\;0.402\;\left(r^{2}\;=\;0.989,\;n\;=\;9\right).$$

$$m_{\text{Ma5Vc}}=\;-0.055\;\times \;\left[{\text{g L}}^{-1}\text{NaCl}\right]\;+\;0.396\;\left(r^{2}\;=\;0.995,\;n\;=\;9\right).$$

$$m_{\text{MaAVc}}\;=\;-0.042\;\times \;\left[{\text{g L}}^{-1}\text{NaCl}\right]\;+\;0.424\;\left(r^{2}\;=\;0.950,\;n\;=\;12\right).$$

Strains Ma1Vc and Ma5Vc showed a lethal dose value of 7 gL^‐1^ NaCl, whereas the value for the MaAVc strain was 10 gL^‐1^ NaCl, as observed previously for this strain (Melero‐Jiménez et al., 2019).

### 3.2 The limit of resistance to salinity under slow and fast deterioration rates

**FIGURE 4:**
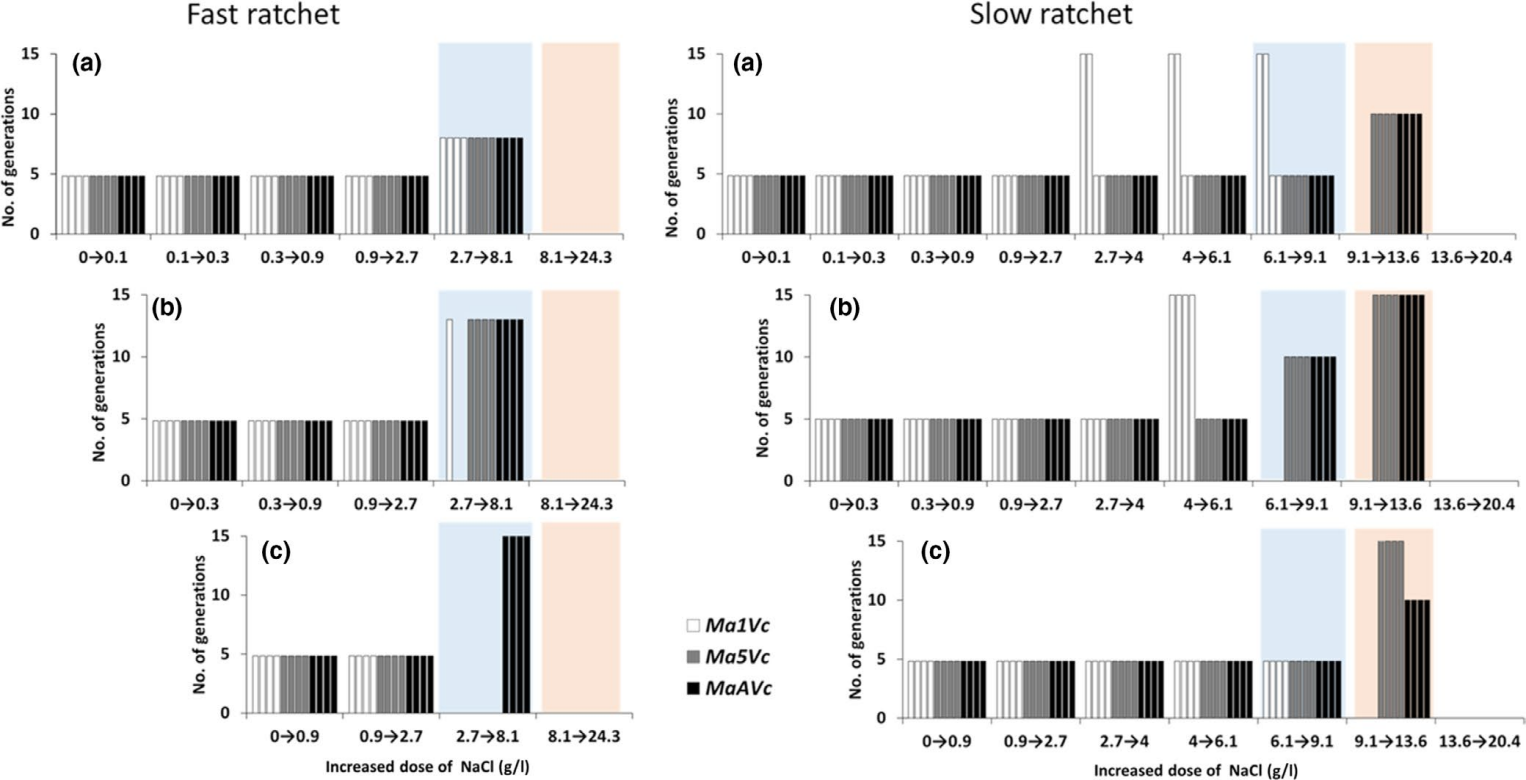
Number of generations required by the cultures of Ma1Vc, Ma5Vc and MaAvc strains of *Microcystis aeruginosa* to grow under increasing doses of NaCl during the ratchet experiments. The fast ratchet is depicted in the left panel and the slow ratchet on the right panel. The letters (a, b, and c) represent different initial salinity doses used at each ratchet experiment (0.1, 0.3, 0.9 gL^‐1^ NaCl respectively). Four independent cultures (columns) were tested per each strain (different column colour). The concentration increment that includes the initial lethal doses for wild‐type strains is indicated by blue (Ma1Vc and Ma5Vc) and orange (MaAVc) background

Under fast salinity increase, no differences were observed in the limit of resistance of the three strains, 8.1 gL^‐1^ NaCl being the highest concentration at which cells could proliferate (Figure 4). However, the limit of resistance achieved by strains Ma1Vc and Ma5Vc was determined by the initial salinity dose at which the ratchet experiment started, as they were not be able to grow at 8.1 gL^‐1^ NaCl, when the initial dose was 0.9 gL^‐1^ NaCl. For example, in the case of the strain Ma1Vc, the number of populations (replicates) able to grow up to 8.1 gL^‐1^ NaCl was 4, 1 and 0 when the initial doses were 0.1, 0.3, and 0.9 gL^‐1^ NaCl, respectively (Figure 4). The limit of resistance observed was higher when the rate of salinity increase was slow. Furthermore, the limit of resistance differed among the strains (9.1 gL^‐1^ NaCl for Ma1Vc, and 13.6 gL^‐1^ NaCl for Ma5Vc and MaAVc) and a greater number of replicate cultures surpassed their initial lethal dose (Table 1). Besides, the number of generations required to reach the lethal dose was lower than that found for the fast ratchet (Figure 4). However, in both ratchet experiments, the initial concentration determined the number of cultures that reached the initial lethal dose and the number of generations needed. Finally, inter‐replicate variation was detected, as some replicates of strain Ma1Vc required different numbers of generations to be able to grow at the next NaCl level during the slow ratchet experiment (Figure 4).

**TABLE 1:** Increase of the tolerance to salinity with respect to the initial lethal dose, expressed as a percentage, for the three *M. aeruginosa* strains after running a slow or fast ratchet protocol (see Figure 4)

Strain	Increased tolerance (%)	
	Slow ratchet	Fast ratchet
Ma1Vc	30 (8)	11 (5)
Ma5Vc	94 (12)	11 (8)
MaAVc	36 (12)	0 (12)

Note

The number of cultures (from the initial 12) that were able to grow above the initial lethal dose, are shown in brackets.

### 3.3 Acclimation versus adaptation

In order to disentangle whether salinity resistance was supported by acclimation or adaptation, the growth rates (*m*) of the cells that showed tolerance to the highest salinity level were computed. The *m* values in NaCl free medium of the ancestral wild‐type cells were 2.2–2.6 times higher than those found in the salinity‐resistant cells, in the three strains tested (Table 2). However**,** no significant differences were detected between the *m* values of the salinity‐resistant cells of all strains, cultured first in the absence of NaCl additions and later at the highest salinity level they could tolerate (Table 2). This result points out to adaptation as the process involved in the salinity resistance of the cells derived from the ratchet experiment.

**TABLE 2:** Growth rate (*m*) of the ancestral, wild‐type cells in BG11‐50% medium, and of the salinity‐resistant cells obtained after the ratchet experiments, grown for 15 days in BG11‐50% and after being transferred for another 15 days to BG11‐50% supplemented with 9.1 gL^‐1^ NaCl (Ma1Vc strain) or 13.6 gL^‐1^ NaCl (Ma5Vc and MaAVc strains)

Strain	*m* (d^−1^)			*H* test	*p‐*value
	Wild‐type cells	Resistant cells (BG11‐50%)	Resistant cells (BG11‐50% + NaCl)		
Ma1Vc	0.41 ± 0.01 a	0.18 ± 0.02 b	0.18 ± 0.01 b	6.215	.044
Ma5Vc	0.39 ± 0.01 a	0.18 ± 0.02 b	0.17 ± 0.03 b	6.845	.032
MaAVc	0.45 ± 0.01 a	0.17 ± 0.02 b	0.17 ± 0.01 b	6.385	.041

Note

Different letters indicate significant differences (Mann–Whitney test) among *m* values. Data are mean ± *SD* (*n* = 3 and 4 for wild‐type and salinity‐resistant cells, respectively).

## 4 DISCUSSION

Does the rate of environmental change modulate the limit of resistance to salinity? We found that the cultures of *M. aeruginosa* exposed to a slow rate of salinity increase were the least likely to become extinct and showed a higher limit of resistance to salinity than the cultures exposed to a fast rate of salinity increase. This is in agreement with other experimental studies, which also demonstrated that slow rates of environmental change lead to less extinction, or better adapted populations (Collins & de Meaux, 2009; Perron et al., 2008,2006; Bell & Gonzalez, 2011; Killeen et al., 2017). In fact, in most of the experimental populations, the number of generations required to observe survival at a salinity level just above the initial lethal dose was lower when the rate of salinity increase was slow (around 5–10 in the slow rachet versus 10–15 in the fast ratchet). A similar result was found in the chlorophycean *Chlamydomonas* exposed to selective conditions (Collins & de Meaux, 2009), as well as in yeasts and bacteria (Bell & Gonzalez, 2011; Gonzalez & Bell, 2013; Lindsey et al., 2013; Perron et al., 2008,2006; Samani & Bell, 2010).

In this study, we provide an empirical demonstration of how the future evolutionary trajectory of an organism could be determined by the rate of environmental change to which it has been exposed, in particular, we addressed the differential adaptive potential of *M. aeruginosa* to different rates of salinity increase. Theoretically, when a population is exposed to a stress situation, individuals with the highest fitness in the new environment persist and the most sensitive ones would be eliminated by natural selection (Bell, 2017). The recovery of the population would be determined by the speed and severity of environmental deterioration (Bell & Collins, 2008). If the severity is not enough to eliminate the entire population, a selection of the most resistant organisms will occur. However, once this initial stress has been overcome, survival would be determined by the rate of deterioration (Bell & Collins, 2008). Most studies have traditionally focused on the range of tolerance to a selective agent rather than to explore the limit of resistance (i.e., when the initial lethal dose is exceeded). However, knowledge of the limit of resistance of cyanobacteria is essential because they play a critical role in aquatic ecosystems (Kirk, 1994).

Ratchet experiments showed that *M. aeruginosa* can proliferate at salinity levels up to 9.1–13.6 gL^‐1^ NaCl, depending on the strain. The value for strain MaAVc, 13.6 gL^‐1^ NaCl, was slightly lower than the limit of resistance observed in another ratchet experiment with the same strain (15.1 gL^‐1^ NaCl; Melero‐Jiménez et al., 2019); however, the concentration increase used was different, so the results are not strictly comparable. It is likely that the limit of resistance could be even higher under natural conditions, due to the vastly greater number of cells in natural populations and the possibility of forming colonies, which could protect the cells from the external environment (Liu, Huang, & Qin, 2018).

The ratchet protocol cannot distinguish whether acclimation or adaptation, or both processes, occurred during the experiment. However, the results of the complementary experiment showed that the growth rate of the most resistant cells of each strain obtained in the slow ratchet experiment was similar when grown for more than seven generations first in the absence of NaCl additions and later in the presence of high NaCl concentrations (Table 2). This result is an evidence to support the hypothesis that genetic mechanisms would be involved in the survival of these cells at the highest NaCl concentrations reached in the ratchet experiment, that is, that adaptation would explain the observed limit of resistance, although other processes cannot be totally excluded. The same design was performed previously, and it was concluded that the limit of resistance to copper of *M. aeruginosa* was also supported by adaptation (Rouco et al., 2014).

Salinity‐resistant cells, growing in the absence of NaCl, showed a *m* value significantly lower than that found for their wild‐type counterparts under the same conditions (Table 2). It could be hypothesized that in non saline natural environments, salinity‐resistant mutants would be out‐competed by the wild‐type strain. Since mutations are generally deleterious (Keightley & Lynch, 2003), the reduction of the overall mean *m* value is responsible to move the average phenotype of well‐adapted populations further from their optimal value (Bell & Collins, 2008). Mutations usually impose a physiological cost that may affect the survival of adapting populations (Andersson & Levinn, 1999; Coustau, Chevillon, & ffrench‐Constant, R., 2000; Lenski, 1998), and the loss of fitness (estimated by *m* in this study) can be indicated by lower rates of growth and photosynthesis (Bañares‐España et al., 2016). It could be hypothesized that, under future global change scenarios, some *M. aeruginosa* strains, those showing highest adaptation limits, could develop in freshwater ecosystems experiencing increases in salinity or even have a high probability to spread and survive in saline waters. In fact, it has been shown that *M. aeruginosa* could survive for two days in marine waters (Miller et al., 2010).

Results from this work could then provide some insight into the survival of natural populations of *M. aeruginosa* on freshwater ecosystems where salinity levels are increasing. However, we studied only three *M. aeruginosa* strains, so these findings should not be extrapolated to other strains of this species. In fact, the salinity tolerance of *M. aeruginosa* is very variable, from 1 to 14 gL^‐1^ NaCl (Orr et al., 2004; Otsuka et al., 1999; Preece, Hardy, Moore, & Bryan, 2017; Sellner, Lacouture, & Parrish, 1988; Tonk et al., 2007; Verspagen et al., 2006). The high variability in salinity tolerance of natural populations of *M. aeruginosa* could be related with a high genetic variability among strains of this species. For instance, it has been observed that a high percentage of phenotypic variation in several traits in this cyanobacterium is a consequence of genetic variation (Bañares‐España, López‐Rodas, Salgado, Costas, & Flores‐Moya, 2006; Bañares‐España, López‐Rodas, Costas, Salgado, & Flores‐Moya, 2007; López‐Rodas et al., 2006; Rico, Altamirano, López‐Rodas, & Costas, 2006), that is, *M. aeruginosa* shows a high broad‐sense heritability for some traits (Falconer & MacKay, 1996). This could be also the case for salinity tolerance traits, and, consequently, a rapid response to selection by salinity could occur. Recently, Tanabe, Hodoki, Sano, Tada, and Watanabe (2018), Tanabe, Yamaguchi, Sano, and Kawachi (2019) showed that some genes involved in the synthesis of sucrose could be responsible for the salinity resistance of *M. aeruginosa* strains from brackish waters and that their presence could be related with horizontal gene transfer (HGT). Moreover, Des Aulnois et al. (2019) also identified an accumulation of trehalose in freshwater strains grown in high salinity, concluding that the use of one or other solute to maintain osmotic equilibrium could be one of the causes of the difference in salt tolerance of *M. aeruginosa* strains, as well as horizontal gene transfer events. Clearly, more studies to explore the limit of resistance of different *M. aeruginosa* strains isolated from water bodies with diverse salinity levels are still necessary.

Knowledge of the limit of resistance of *M. aeruginosa* to salinity is also relevant from an environmental toxicology point of view (Loftin et al., 2016). In this sense, Tonk et al. (2007) highlighted that salinity fluctuations in brackish waters may not only favor *M. aeruginosa* over other freshwater phytoplankton species, but may also increase the toxin exposure of many aquatic organisms due to the increase of microcystin concentrations. Black, Yilmaz, and Phlips (2011) confirmed that under the influence of substantial salinity inputs, *M. aeruginosa* could potentially survive and may even grow. So, estuaries and nearshore coastal areas where salinity could increase in future scenarios of global change (Paerl & Huisman, 2009; Paerl & Paul, 2012) would face the problem of an increase in microcystin concentration, as the growth of *M. aeruginosa* would not be prevented.

In this study, we tested the limit of resistance of an asexual organism. So, it is important to highlight that the possibility of genetic drift events to occur due to “sampling errors” (Crow & Kimura, 1970) was low during the ratchet experiments, because in each ratchet cycle, the number of cells transferred was 3 × 10^5^ cells. Consequently, the effect of the Hill–Robertson interference is reduced because the population size is large enough to assure the occurrence of new mutations that confer resistance (Huertas et al., 2010; Reboud, Majerus, Gasquez, & Powles, 2007). However, it would be interesting to perform the ratchet protocol with organisms showing sexual reproduction because gene recombination decreases linkage disequilibrium between genes (Otto & Michalakis, 1998). Although sexual reproduction generates more genetic diversity than asexual reproduction (and then a higher possibility to achieve resistance), it would be possible that recombination generates offspring's genotypes showing less fitness than their parentals (Otto & Michalakis, 1998).

In conclusion, we have shown how the rate of environmental change can modulate the limit of resistance of *M. aeruginosa* to salinity and that the limit of resistance to salinity is mainly supported by the selection of new mutant's variants (adaptation), although another mechanisms could be also involved. However, it is necessary to emphasize that these models represent a simplification of natural processes, so more experimental evolutionary studies are necessary to reach a deeper understanding of the organisms’ behavior in nature.

## CONFLICT OF INTEREST

The authors declare no conflicting interests.

## AUTHOR CONTRIBUTION

IJM‐J performed the experiments, analyzed the data, wrote the paper, prepared figures and tables, and reviewed drafts of the paper; EM‐C performed the experiments; MJG‐S and AF‐M conceived and designed the experiments, analyzed the data, contributed reagents/materials/analysis tools, wrote the paper and reviewed drafts of the paper; EBE conceived and designed the experiments, analyzed the data and reviewed drafts of the paper.

## Supporting information

Table S1

Table S2

Table S3

## Data Availability

All relevant data are within the paper and its Supporting Information files (excel files: S1, S2 and S3).
